# Plasma Clusterin and Lipid Profile: A Link with Aging and Cardiovascular Diseases in a Population with a Consistent Number of Centenarians

**DOI:** 10.1371/journal.pone.0128029

**Published:** 2015-06-15

**Authors:** Angela Baralla, Elisabetta Sotgiu, Marta Deiana, Sara Pasella, Sara Pinna, Andrea Mannu, Elisabetta Canu, Giovanni Sotgiu, Antonello Ganau, Angelo Zinellu, Salvatore Sotgia, Ciriaco Carru, Luca Deiana

**Affiliations:** 1 Department of Biomedical Sciences, University of Sassari, Sassari, Italy; 2 Associazione "L’Isola dei Centenari", Sassari, Italy; 3 Department of Clinical and Experimental Medicine, Cardiology Unit, University of Sassari, Sassari, Italy; University College London, UNITED KINGDOM

## Abstract

The role of Clusterin in attenuation of inflammation and reverse cholesterol transfer makes this molecule a potential candidate as a marker for cancer, cardiovascular disease, diabetes mellitus, and metabolic syndrome. In elderly subjects cardiovascular diseases represent the primary cause of death and different clinical studies have shown a positive correlation of these diseases with changes in the lipid pattern. This work aimed at evaluating the relationship between circulating clusterin and the biochemical parameters that characterize the lipid profile of a Sardinian population divided into five age groups including centenarians; the high frequency in Sardinia of these long-lived individuals gave us the opportunity to extend the range of the age groups to be analyzed to older ages and to better evaluate the changes in the lipid balance during ageing and its relationship with clusterin concentration in plasma. Our results showed that Clusterin concentration values of the youngest group were more similar with the centenarian’s group compared to the other age groups, and a positive correlation arises with LDL. Furthermore given the high prevalence of cardiovascular diseases in the population examined and the association of Clusterin with these pathologies we evaluated Clusterin concentration variation in two groups with or without cardiovascular diseases. In presence of cardiovascular disease, Clusterin is significantly related to the most atherogenic components of lipid profile (total cholesterol and LDL), especially in women, suggesting its potential role in modulating cardiovascular metabolic risk factors.

## Introduction

Clusterin (CLU)/Apo J is a glycoprotein of 70–80 kDa, it is a disulfide linked heterodimer composed of α and β subunits generated by a single cleavage from the protein precursor. It was originally identified in ram rete testis fluid, and later on it was found to be expressed in several tissues and in all human fluids [1]. It is present in plasma as a soluble protein or as a component of a lipid-poor subclass of high-density lipoproteins (HDLs) of molecular mass of 70–200 kDa [2,3]. Recent proteomic analyses revealed that CLU is also bound to LDL and very LDLs [4–6]even if the role in these lipoproteins is unknown. Clusterin has been reported to be implicated in several physiological processes such as sperm maturation, lipid transportation, complement inhibition, tissue remodeling, membrane recycling, cell-cell and cell-substratum interaction, stabilization of stressed proteins in a folding-competent state and promotion or inhibition of apoptosis. CLU is ubiquitously expressed in most cells and tissues, and is upregulated under a variety of pathological conditions including ageing, diabetes, atherosclerosis, degenerative diseases [7–9], moreover it has recently drawn much attention because of its association with cancer promotion and metastasis [10–12]. CLU can exist within the cell to function in either proapoptotic or prosurvival processes. This diverse set of functions can be attributed to the existence of two alternatively spliced forms of the *Clu* gene that encode secretory CLU (sCLU) or nuclear CLU (nCLU). The sCLU form seems to be cytoprotective [13] while nCLU migrates to the nucleus on cytotoxic stress to trigger cell death [14,15]. Although its exact function has not been clearly defined, there is increasing evidence that CLU is induced by stress and functions as a cytoprotective extracellular chaperone similar to small heat shock proteins [16–18]. During in vivo ageing *Clu* gene expression was found to increase from gestation to adults in humans [19], in the human pituitary gland [20], in the rat ventral prostate [21], in human glial cultures [22] and in human lymphocytes [23]. In serum sCLU levels seems to increase during in vivo ageing at least in males [8]. During vascular damage sCLU was found to accumulate in the human serum of diabetes type II patients or during myocardial infarction [8]; sCLU accumulates in the artery wall during atherosclerosis [24] and is present in human and mouse atherosclerotic lesions, but not in normal arterial tissue [25,26]. In tissue cultures it acts as an acceptor of cholesterol from macrophage-derived foam cells suggesting that CLU, present at high concentrations around stressed cells, has the potential to remove cholesterol from damaged cell membranes and then deliver it to HDL particles for subsequent disposal through reverse cholesterol transport [27]. In another work ApoJ was find to co-localize with E-LDL in atherosclerotic lesions and may thus subserve protective functions through its capacity to inactivate C5b-9 complement complexes and by reducing the cytotoxic effects of modified LDL on cells that gain contact with the lipoprotein [28]. Recent experiments by Martinez-Bujidos et al., performed with CLU-depleted LDLs demonstrated that CLU plays an active protective role against LDL aggregation [29]. Moreover in a recent work fasting plasma clusterin levels were found to be positively correlated with BMI, a parameter of whole body adiposity in healthy adults [30]. The purpose of this study was to evaluate clusterin concentration in plasma samples of a Sardinian population divided into five age groups with a consistent number of centenarians, as well as the concentration of the biochemical parameters that characterize the lipid profile of an individual, including high-density lipoprotein (HDL), low-density lipoprotein (LDL), triglycerides and total cholesterol, in order to establish if any relationship can be found between circulating clusterin and those parameters. Furthermore, since almost half of the population analysed suffered from cardiovascular diseases, which are the most common diseases between elderly people, and considering that several studies hold that CLU is implicated in various cardiovascular diseases (e.g. atherosclerosis, coronary heart disease and myocardial infarction) [26,31–34] the same correlation analyses mentioned above with lipid parameters have been evaluated for CLU dividing the population by people with or without cardiovascular diseases.

## Materials and Methods

### Study Population

Subjects for this study were recruited through the AKeA project that studies the Sardinian centenarians [35,36]. The AKeA project has been approved by the Bioethic Committee of the University of Sassari and ASL N°1 Sassari. The major criteria for the selection of the partecipants were the chronological age, the residence in Sardinia (the majority of them being people coming from the same villages of centenarians) and the apparently healthy status. An informed written consent, approved by the above mentioned Bioethic Committee, was obtained from each subject or from a legally responsible family member. A standardized questionnaire on general information was also administered concerning gender, age, smoking habits, alcohol drinking, medications and health status (Table 1). Smoking was defined as smoking at least one cigarette a day and lasting more than six months. Alcohol drinking was defined as drinking any type of alcoholic beverage at least once a week and lasting more than six months. Smoking and drinking were classified as current (yes) previous (ex smoker/drinker) or never (no smoker/drinker). Hypertension was defined according to repeated blood pressure measurements ≥ 140/90 mmHg or previous diagnosis of hypertension and antihypertensive treatment. Almost half of the population (47.2%) had cardiovascular diseases which is the most common disease between elderly people (cardiovascular diseases were grouped as arterial hypertension (41.5%), angina (4.5%), heart failure (4.1%), myocardial infarction (1.2%), other cardiac diseases (1.6%; valvular diseases or cardiomyopathies)) while a minority of them suffered from chronic conditions (5.3%, epatic 15.8%, respiratory 11.4%, nervous systems 7.3%, tumors 8.5%, endocrine metabolic disorders 25.6%, cerebrovascular diseases 7.7%). Table A in S1 File shows the same data stratified by age groups. The population with 264 subjects was divided into 5 age groups (20–50 years (y): N = 53, 60–75y N = 49, 80–89y N = 48, 90–99y N = 46, ≥100y N = 68) for the age related analysis, while to examine the relationship with cardiovascular diseases the population was divided into two groups with or without cardiovascular diseases (N = 116, N = 148, respectively).

**Table 1:** Medications, smoking and drinking habits of the population analysed.

	Age group				
	20–50 years (N = 53)	60–75 years (N = 49)	80–89 years (N = 48)	90–99 years (N = 46)	100–196 years (N = 68)
**Medications**					
**Β-blockers (%)**	**1.4**	**0**	**58.5**	**31.3**	**32.4**
**ACE-inhibitors (%)**	**1.4**	**0**	**5.7**	**0**	**0**
**Ca-inhibitors (%)**	**0**	**0**	**26.4**	**16.7**	**14.7**
**Angiotensin II-antagnists (%)**	**0**	**2**	**15.1**	**10.4**	**14.7**
**Vasodilators (%)**	**0**	**0**	**11.3**	**4.2**	**0**
**Diuretics (%)**	**0**	**0**	**13.2**	**6.3**	**4.4**
**Statins (%)**	**0**	**4**	**20.8**	**18.8**	**14.7**
**Antiplatelet drugs**	**0**	**0**	**3.8**	**0**	**0**
**Smokers %**					
**No**	**54.5**	**87.5**	**63.5**	**61.7**	**71.2**
**Yes**	**36.4**	**12.5**	**3.8**	**8.5**	**6.1**
**Ex**	**9.1**	**0.0**	**32.7**	**29.8**	**22.7**
**Drinkers (%)**					
**No**	**92.3**	**12.5**	**3.8**	**8.5**	**6.1**
**Yes**	**7.7**	**0.0**	**32.7**	**29.8**	**22.7**
**Ex**	**0.0**	**66.7**	**46.2**	**42.6**	**24.2**

### Biochemical Parameters

Blood samples were collected in EDTA-coated tubes for clusterin analyses, and processed within two hour. The red cell fraction was then separated by centrifugation at 2500g at 4°C for 15 min and the clear plasma supernatant was stored in aliquots frozen at -80°C. For cholesterol, LDL, triglycerides and HDL blood was collected into gel vacutainer tubes and their concentration were assessed through enzymatic method using the ARCHITECT cSystems. Creatinine, Glucose and Urea with the ARCHITECT c8000 using enzymatic methods. For clusterin concentration in plasma a competitive ELISA kit (AdipoGen, Korea) was used following manufacturer instructions. All the statistical analyses were performed using SPSS v21 and MedCalc v12.7 softwares. The differences between groups were assessed through the Kruskal Wallis test followed by pairwise comparison Dunn’s test; for the correlation analyses the Spearman test was used. A p-value< 0.05 was considered statistically significant.

## Results

**Fig 1:**
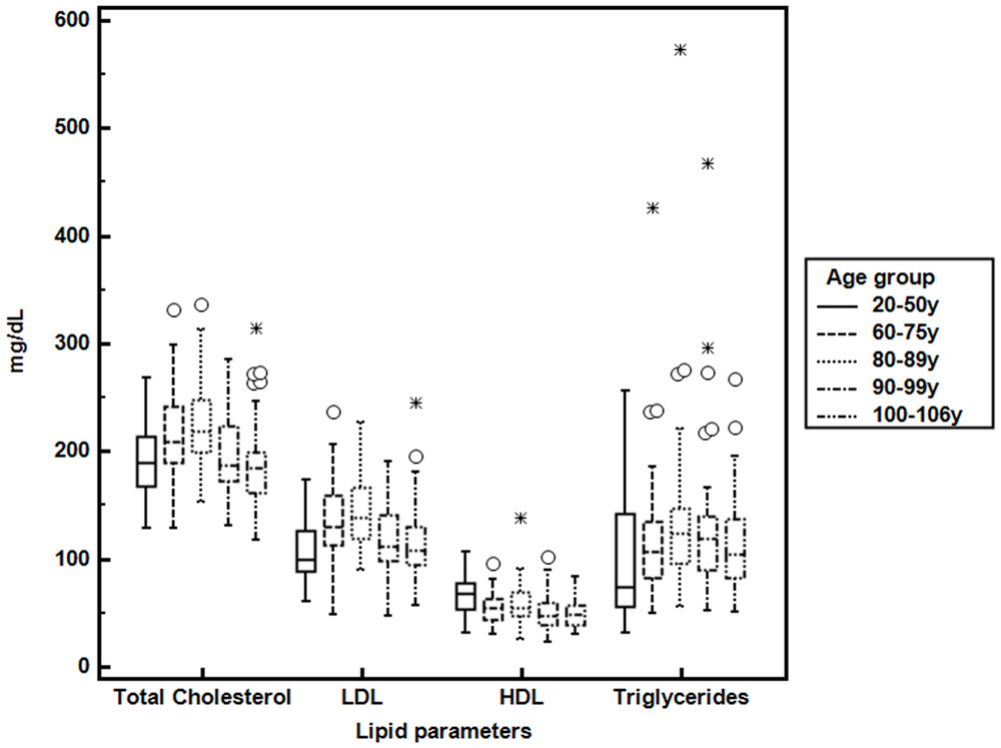
Lipid parameters’ values in the five age groups. Boxplots represent the Lipid parameters analysed in the five age groups, total cholesterol, LDL (low density lipoprotein), HDL (high density lipoprotein) and Triglycerides. Circles represent outliers and asterix extreme outliers.

**Fig 2:**
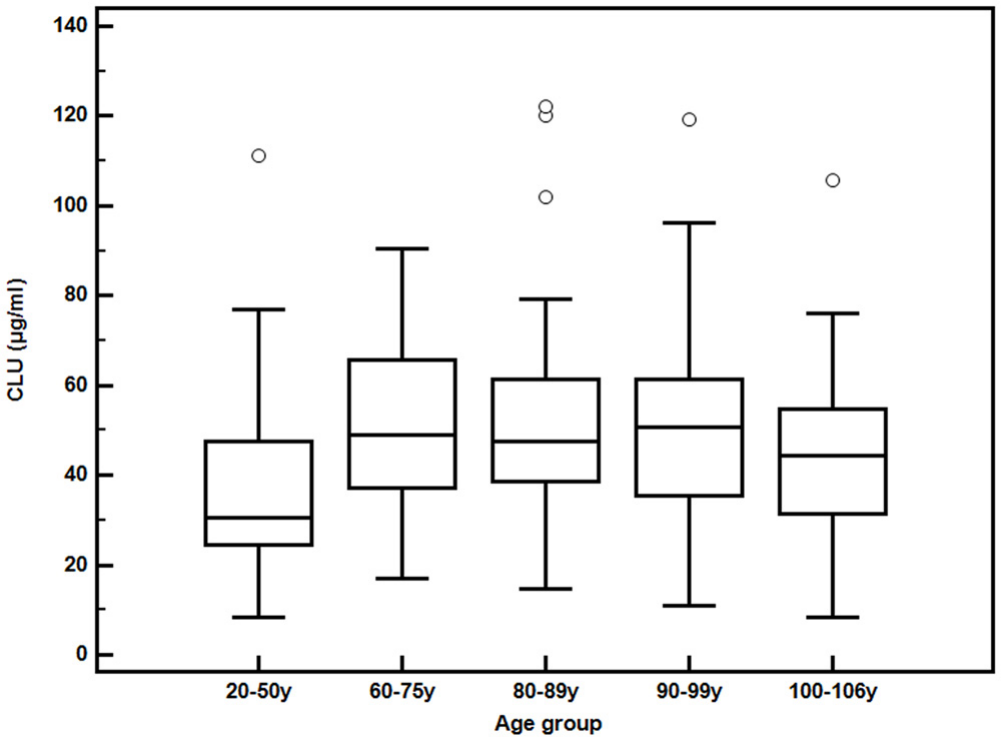
Clusterin concentration values in the five age groups. Boxplots represent the Clusterin concentration values in the five age groups. Circles represent outliers.

In this paper we wanted to evaluate if Clusterin concentration changes during ageing and if a relationship exists with changes in the biochemical parameters characterizing the lipid profile of an individual such as high-density lipoprotein (HDL), low-density lipoprotein (LDL), triglycerides and total cholesterol. Table 2 shows the values of the parameters mentioned above in the population analyzed divided by age groups as previously described and the significant differences highlighted by Kruskal Wallis test. This analysis revealed a significant change in concentration for all of the parameters analyzed. Cholesterol, LDL and Triglycerides had an higher value in the group of 80–89years while in the older groups their level was lower (Table 2, Fig 1). HDL had the highest value in the group of 20–50 years with lower values in the other four groups. Clusterin had higher values in the group of 90–99 years while the group of centenarians had CLU values lower compared to the other elderly groups (Table 2, Fig 2), the pairwise comparisons test in the Supplementary data shows the p-values relative to the comparisons by pair groups (Table B in S1 File) highlighting how the youngest group has clusterin values statistically more similar to the centenarian’s group. No significative differences emerged between the five age groups for the other biochemical parameters evaluated (glucose, urea and creatitìnine) (Table 2). Considering the whole population the correlation analyses with the Spearman’s test showed that clusterin is positively correlated with LDL cholesterol (ρ = 0.196 p = 0.002) (Table 3) and the evaluation of gender differences revealed statistically significant correlations only in the females group, in particular a positive correlation between CLU and LDL (ρ = 0.29, p = 0.001) and a negative correlation between CLU and HDL (ρ = -0.183, p = 0.031) (Table 3). Dividing the population in the five age groups the correlation between CLU and LDL is concentrated only inside the group of 90–99 years old (ρ = 0.403, p = 0.006) (Table 4). Since the population analyzed had an high number of individuals with cardiovascular diseases and considering the association of Clusterin with different pathologies related to cardiovascular diseases we wanted to see if any difference existed between the two groups in the concentration values of both clusterin and lipid parameters. Table 5 shows how only triglycerides values were significantly different. For concentration values divided by age with or without cardiovascular diseases and relative p-values for comparisons see Table C in S1 File. The correlation analyses showed a stronger correlation with LDL (ρ = 0.30 p = 0.002) and other two correlations emerged, with total cholesterol and with triglycerides (ρ = 0.258 p = 0.007, ρ = 0.234 p = 0.014) but only inside the group of people that had cardiovascular diseases, while no significant correlation came out inside the healthier group (Table 5). When considering gender differences only females with cardiovascular diseases showed a positive correlation between CLU and LDL (ρ = 0.432 p = 0.001) and with total cholesterol (ρ = 0.406 p = 0.001) (Table 6).

**Table 2:** Clusterin and biochemical parameter’s concentrations by Age group and Kruskal Wallis test for comparisons between the five age groups.

	Age group										
	20–50 years (N = 53)		60–75 years (N = 49)		80–89 years (N = 48)		90–99 years (N = 46)		100–196 years (N = 68)		K-W test^c^
	Median	IQR^a^	Median	IQR^a^	Median	IQR^a^	Median	IQR^a^	Median	IQR^a^	p-value
**Clusterin (μg/ml)**	30.42	(24.34–48.29)	48.94	(37.12–64.86)	47.52	(38.64–60.96)	50.59	(35.52–61.40)	44.37	(31.45–55.41)	<0.0001
**Total Cholesterol (mg/dL)**	189	(168–214)	209	(190–241)	218.50	(199–258)	186.50	(172–223)	184.50	(162–199.5)	<0.0001
**HDL (mg/dL)** ^b^	68	(54–78)	55	(44–63)	55	(47.5–69.5)	47.50	(38.5–60)	49	(39–57)	<0.0001
**LDL (mg/dL)** ^b^	99.0	(89.2–126)	130.3	(113–159.5)	138.50	(119–166.5)	112.0	(99–140)	107.8	(95–130)	<0.0001
**Triglycerides (mg/dL)**	74	(56–141)	107	(83–132)	123.5	(96.5–146.5)	119.5	(90–140)	105	(82–138)	0.001
**Glucose (mg/dL)**	83	(73–93)	87	(84–99)	85	(58–95)	87.5	(79–108)	91	(82–110)	0.062
**Urea (mg/dL)**	4.3	(3.6–5.3)	4.6	(4.1–5.6)	5.7	(3.9–6.3)	5.3	(3.45–7.10)	5	(3.7–6.4)	0.329
**Creatinine (mg/dL)**	0.8	(0.75–0.9)	0.80	(0.7–0.9)	1.10	(0.85–1.3)	1	(0.7–1.6)	1	(0.9–1.3)	0.0001

^a^ IQR, interquartile range

^b^HDL, high density lipoprotein cholesterol; LDL, low density lipoprotein cholesterol

^c^K-W = Kruskal Wallis test

**Table 3:** Correlations between Clusterin and Lipid parameters in the whole population and divided by gender.

	Clusterin					
	All (N = 264)		Gender			
			Male (N = 123)		Female (N = 141)	
	ρ^a^	p-value	ρ^a^	p-value	ρ^a^	p-value
**Total Cholesterol**	0.087	0.159	0.073	0.429	0.124	0.142
**HDL** ^b^	-0.117	0.062	-0.037	0.695	-0.183	0.031
**LDL** ^b^	0.196	0.002	0.099	0.302	0.290	0.001
**Triglycerides**	0.073	0.240	0.124	0.176	0.038	0.656

^a^ρ, Spearman's rank correlation coefficient

^b^HDL, high density lipoprotein cholesterol; LDL, low density lipoprotein cholesterol;

**Table 4:** Correlation between Clusterin and Lipid parameters divided by Age groups.

	Clusterin									
	Age group									
	20–50y ((N = 53)		60–75y (N = 49)		80–89y (N = 48)		90–99y (N = 46)		100–106y (N = 68)	
	ρ^a^	p-value	ρ^a^	p-value	ρ^a^	p-value	ρ^a^	p-value	ρ^a^	p-value	
**Total Cholesterol**	-0.322	0.069	0.117	0.429	0.105	0.476	0.243	0.104	-0.116	0.351
**HDL** ^b^	-0.137	0.327	-0.084	0.575	-0.128	0.409	0.059	0.702	-0.026	0.839
**LDL** ^b^	-0.255	0.065	0.159	0.287	0.227	0.138	0.403	0.006	-0.112	0.401
**Triglycerides**	-0.102	0.469	0.096	0.518	-0.035	0.812	-0.070	0.644	0.070	0.575

^a^ρ, Spearman's rank correlation coefficient

^b^LDL, low density lipoprotein cholesterol; HDL, high density lipoprotein cholesterol;

**Table 5:** Clusterin and lipid parameters concentrations by presence or absence of cardiovascular diseases and Kruskal Wallis test for comparisons between the two groups.

	Cardiovascular diseases				
	No (N = 148)		Yes (N = 116)		K-W test^c^
	Median	IQR^a^	Median	IQR^a^	p-value
**Clusterin (μg/ml)**	44.84	(32.99–60.97)	46.87	(36.02–58.88)	0.244
**Total Cholesterol (mg/dL)**	194.50	(173–218.5)	201.00	(177–231)	0.359
**HDL (mg/dL)** ^b^	52.00	(43–64)	52.00	(42–63)	0.626
**LDL (mg/dL)** ^b^	116.50	(97.5–140.5)	122.00	(102–143)	0.614
**Triglycerides (mg/dL)**	99.50	(75–126)	119.00	(90–152)	0.001

^a^ IQR, interquartile range

^b^HDL, high density lipoprotein cholesterol; LDL, low density lipoprotein cholesterol

^c^K-W = Kruskal Wallis test

**Table 6:** Correlation between Clusterin and Lipid parameters in the two groups with or without cardiovascular diseases and divided by gender.

	Clusterin											
	Cardiovascular diseases				Cardiovascular diseases by gender							
	no (N = 148)		yes (N = 116)		no (male) (N = 62)		no (female) (N = 86)		yes (male) (N = 56)		yes (female) (N = 60)	
	ρ^a^	p-value	ρ^a^	p-value	ρ^a^	p-value	ρ^a^	p-value	ρ^a^	p-value	ρ^a^	p-value
**Total Cholesterol**	-0.027	0.770	0.258	0.007	0.042	0.745	-0.044	0.734	0.100	0.487	0.406	0.001
**HDL** ^b^	-0.086	0.353	0.037	0.706	-0.014	0.913	-0.148	0.264	0.036	0.811	0.006	0.964
**LDL** ^b^	.0052	0.582	0.300	0.002	0.035	0.800	0.115	0.380	0.151	0.311	0.432	0.001
**Triglycerides**	-0.066	0.470	0.234	0.014	0.017	0.898	-0.118	0.367	0.236	0.098	0.224	0.086

^a^ρ, Spearman's rank correlation coefficient

^b^HDL, high density lipoprotein cholesterol; LDL, low density lipoprotein cholesterol

## Discussion

The role of clusterin in attenuation of inflammation and reverse cholesterol transfer makes this molecule a potential candidate as a marker for cancer, cardiovascular diseases, diabetes mellitus, and metabolic syndrome. In elderly subjects cardiovascular diseases represent the primary cause of death. Different epidemiological and clinical studies have shown that the incidence of atherosclerosis related vascular diseases is positively correlated with changes in the lipid pattern [37,38] highlighting the importance of cardiocirculatory conditions on human survival. The results obtained for Clusterin concentration in our population agreed in part with the data shown in literature by the work of Trougakos et al., where an increase of this protein during ageing occurs until 90 [8,31]. The novelty of this work is that our investigations spanned over older people showing an increase even after 90 years, but in centenarians, which have a life-long survival advantage [29,30], the clusterin values are lower compared to the other elderly groups. Until now, only one work has focused the attention on clusterin expression in centenarians [39], in particular Trougakos et al., found that clusterin gene expression level in lymphocyte samples of 25 centenarians was reduced as compared to elderly donors. Our work highlights for the first time the reduction of clusterin in plasma samples of centenarians at the protein level and fill the gap in the age range 90–99years. This behavior can be related, as already hypothesized by Trougakos et al.[39], to the clusterin function as a sensitive biosensor of environmental insults and particularly oxidative stress. Centenarians are considered as a model of successful ageing characterized by low ROS (reactive oxygen species) load [40], so, its reduction can be attributable to the lower ROS load of centenarians. Determination of oxidative stress biomarkers in plasma together with clusterin levels can be important to support this interesting hypothesis raised by Franceschi et al. Considering the two groups with or without cardiovascular diseases no statistically significant difference in clusterin concentration values were detectable. CLU involvement in reverse cholesterol transport and its potential protective role in atherogenesis through its binding to enzymatically modified low-density lipoprotein and its capacity to reduce fatty acid mediated cytotoxicity lead us to investigate the relationship of this protein with the lipid profile of the population analyzed. These analyses highlighted a positive correlation between Clusterin and LDL cholesterol. Dividing the population by gender the positive correlation appears only in the females group with an higher correlation value than the one detected for the whole population; furthermore, a negative association with HDL was found in this group. The association between CLU levels and LDL has been demonstrated for the first time by Aronis et al. in a population of young healthy individuals [41], but this group found a positive correlation even for total cholesterol. This work confirms the positive correlation found by Aronis et al. for LDL cholesterol extending the result to an higher age range. Moreover it highlighted gender differences revealing a stronger correlation for females between Clusterin and LDL-cholesterol. It has been reported that androgen, a member of sex hormone influences clusterin secretion [42], hence, hormone regulation can be a reason for the gender differences observed in the present work. Dividing the population in the five age groups the correlation between CLU and LDL is concentrated only inside the group of 90–99 years old. One reason for not having found a significant correlation in the other age groups can be related the small sample size. Ageing is a multifactorial process modulated by the interplay among genetic and environmental factors [43] and is associated with multiple, systemic dysfunctions of the body and accompanied by lipid metabolism disorders that in turn lead to more chronic medical conditions [44]. One of the leading cause of death in the elderly population are cardiovascular diseases and the association with dyslipidemia rises substantially with advancing age [45]. For this reason and considering the high number of people with cardiovascular diseases in our sample population, (see materials and methods for details on how they have been gathered) we made separate analyses for the presence or absence of cardiovascular diseases. The results of theses analyses showed a positive correlation between CLU and LDL only in the group with cardiovascular diseases and stronger than in the whole population. Moreover other lipid parameters besides LDL (total cholesterol and triglycerides) came out to be significantly correlated to CLU. In a recently published paper Martinez-Bujidos et al., provided evidence that CLU plays an active protective role against LDL aggregation and they proposed that the increased expression of CLU in atherosclerotic lesions could be a response of the arterial walls to LDL aggregation, the initial step of atherosclerosis [29]. These findings could be a possible explanation to the positive correlation obtained between CLU and LDL only inside the group with cardiovascular diseases if we consider that CLU changes can be the result of its action of counteracting the proatherogenic function of LDL through a prevention of their aggregation. The correlations highlighted by this work suggest a gender dependent lipid metabolism regulation in which clusterin protein may be involved and hence supports the hypothesis of its role in the disease mechanisms. Further investigations would be addressed to elucidate the molecular mechanisms involved in such regulation. During ageing usually an increase in total cholesterol levels occurs in both genders, while in advanced aging (octogenarians, nonagenarians), people show a lipid profile typical of low risk of atherosclerotic disease [46], with high HDL, low total cholesterol and low LDL and high HDL/total cholesterol ratio [47,48] compared with septuagenarians [48,49]. Epidemiological studies found that the plasma levels of HDL cholesterol decrease with increasing age, but in the elderly they are unchanged or slightly increased [50]. A paper by Baggio et al. reported that the mean HDL levels of centenarians (both males and females) are 20% lower than those of 65-year-old subjects [51]. The decreased levels of HDL can be due to inflammatory, hormonal and metabolic changes which are more prevalent with aging, while a possible explanation to the higher levels of HDL seen in some population in elderly people can be due to the decreased levels of androgens in aging men which result in an increase of HDL particles due to the reduced catabolism driven by testosterone [44]. In our sample population there is an evident change in the lipid profile with significant differences in the five age groups for all lipid parameters (Table 2). In our population an increase of total cholesterol and LDL cholesterol occurs from 20 to 89 years, while in nonagenarians and centenarians their level decreases but at values that are considered to be in the normal range (186.5mg/dL and 184mg/dL for nonagenarians and centenarians respectively) while different studies have reported that very low levels of total cholesterol are associated with an all-cause mortality [52,53]. The low values of total cholesterol and LDL can be important in reaching a lipid balance capable of preventing the occurrence of cardiovascular diseases. HDL levels, whose higher values are usually correlated with lower cardiovascular risk, were slightly lower if compared to the other groups. but at the same time the concentration values were above 40mg/dL which is desirable according to the ATP III guidelines [54]. In conclusion, the positive correlation found between Clusterin and the most atherogenic components of lipid profile (total cholesterol and LDL), highlights the importance that this protein can have in modulating the lipid balance of an individual especially during aging where visible changes in the lipid parameters occur, and in cardiovascular diseases where the absolute risk associated with dyslipidemia rises with advancing age [45].

## Supporting Information

S1 File Distribution of the diseases by age group in the population analyzed (Table A). Paiwise comparisons between the five age groups (Table B). Clusterin and lipid parameters concentrations divided by presence or absence of Cardiovascular diseases and by age groups (Table C).
(DOC)
