# Supplementary material for: Plasma Clusterin and Lipid Profile: A Link with Aging and Cardiovascular Diseases in a Population with a Consistent Number of Centenarians
Source: PLoS One. 2015 Jun 15;10(6):e0128029. doi: 10.1371/journal.pone.0128029 (PMC4468059; doi:10.1371/journal.pone.0128029)
Supplement: S1 File — Paiwise comparisons between the five age groups (Table B). Clusterin and lipid parameters concentrations divided by presence or absence of Cardiovascular diseases and by age groups (Table C). (DOC) [file pone.0128029.s001.doc]

**S1 Table A.** Distribution of the diseases by age group in the population analyzed

| **Diseases** | **Age group** | | | | | | | | | |
| --- | --- | --- | --- | --- | --- | --- | --- | --- | --- | --- |
|  | **60-75y (N=20)** | | **80-89y (N=34)** | | **90-99y (N=26)** | | **100-106y (N=28)** | | **60-75y (N=20)** | |
| **Tumors (%)** |  |  | 1 | 2.0 | 12 | 23.1 | 2 | 4.2 | 6 | 9.5 |
| **Endocrine metabolic disorders (%)** | 8 | 23.5 | 13 | 26.5 | 22 | 42.3 | 10 | 20.8 | 10 | 15.9 |
| **Cerebrovascular diseases (%)** | 1 | 2.9 | 1 | 2.0 | 7 | 13.5 | 4 | 8.3 | 6 | 9.5 |
| **Cardiovascular diseases (%)** | 3 | 8.8 | 20 | 40.8 | 37 | 71.2 | 28 | 58.3 | 28 | 44.4 |
| **Nervous systems diseases(%)** |  |  | 2 | 4.1 | 6 | 11.5 | 2 | 4.2 | 8 | 12.7 |
| **Respiratory diseases(%)** | 2 | 5.9 | 8 | 16.3 | 5 | 9.6 | 7 | 14.6 | 6 | 9.5 |
| **Epatic diseases(%)** | 3 | 8.8 | 6 | 12.2 | 14 | 26.9 | 8 | 16.7 | 12 | 19.0 |
| **Gastrointestinal diseases (%)** | 2 | 5.9 | 2 | 4.1 | 4 | 7.7 | 2 | 4.2 | 3 | 4.8 |

**S1 Table B.** Paiwise comparisons between the five age groups

|  | **Clusterin** | | | | **Total Cholesterol** | | | | **HDL^b^** | | | | |
| --- | --- | --- | --- | --- | --- | --- | --- | --- | --- | --- | --- | --- | --- |
| **Age group** | **20-50y** | **60-75y** | **80-89y** | **90-99y** | **20-50y** | **60-75y** | **80-89y** | **90-99y** | **20-50y** | **60-75y** | **80-89y** | | **90-99y** |
| **60-75y** | <0.001 | - | - | - | 0.051 | - | - | - | 0.004 | - | - | | - |
| **80-89y** | <0.001 | 1 | - | - | <0.001 | 1 | - | - | 0.114 | 1 | - | | - |
| **90-99y** | 0.001 | 1 | 1 | - | 1 | 0.269 | 0.006 | - | <0.001 | 0.988 | 0.127 | | - |
| **100-106y** | 0.1 | 0.794 | 0.636 | 1 | 1 | 0.003 | <0.001 | 1 | <0.001 | 0.252 | 0.017 | | 1 |
|  | | | | | | | | | | | | | |
|  | **LDL^b^** | | | | **Triglycerides** | | | |  | | | | |
| **Age group** | **20-50y** | **60-75y** | **80-89y** | **90-99y** | **20-50y** | **60-75y** | **80-89y** | **90-99y** |  | | | | |
| **60-75y** | <0.001 | - | - | - | 0.262 | - | - | - |  | | | | |
| **80-89y** | <0.001 | 1 | - | - | 0.001 | 0.788 | - | - |  | | | | |
| **90-99y** | 0.264 | 0.562 | 0.017 | - | 0.005 | 1 | 1 | - |  | | | | |
| **100-106y** | 1 | 0.013 | <0.001 | 1 | 0.078 | 1 | 1 | 1 |  | | | | |
| Data are the p-values of the Post-hoc test Dunn’s test applied after the Kruskal Wallis test | | | | | | | | | | | |  | |

S1 Table C. Clusterin and lipid parameters concentrations divided by the presence or absence of Cardiovascular diseases for each age group

|  | **Cardiovascular diseases: no** | | | | | | | | |
| --- | --- | --- | --- | --- | --- | --- | --- | --- | --- |
|  | **Age group** | | | | | | | |  |
|  | **60-75y (N=29)** | | **80-89y (N=14)** | | **90-99y (N=20)** | | **100-106y (N=40)** | | **K-W test^c^** |
|  | **Median** | **IQR^a^** | **Median** | **IQR^a^** | **Median** | **IQR^a^** | **Median** | **IQR^a^** | **p-value** |
| **CLU (µg/ml)** | 41.54 | (35.92 - 60.99) | 50.95 | (38.64 - 69.31) | 58.90 | (38.195 - 66.36) | 45.10 | (29.64 - 52.36) | 0.160 |
| **Total Cholesterol (mg/dl)** | 213 | (200 - 243) | 209 | (177 - 226) | 182 | (173 - 217) | 184 | (162 - 198) | 0.005 |
| **HDL (mg/dl)^b^** | 56 | (45.5 - 62) | 53 | (42 - 64) | 48 | (39 - 61) | 48 | (39 - 54) | 0.113 |
| **LDL (mg/dl)^b^** | 137.4 | (115.6 - 164) | 121.0 | (104 - 178) | 115.0 | (97 - 139.6) | 111.3 | (95 - 127) | 0.008 |
| **Triglycerides (mg/dl** | 110 | (86 - 116) | 124 | (86 - 136) | 112 | (88 - 140) | 100 | (77 - 131) | 0.495 |

|  | **Cardiovascular diseases: yes** | | | | | | | | | |  |
| --- | --- | --- | --- | --- | --- | --- | --- | --- | --- | --- | --- |
|  | **Age group** | | | | | | | | |  |  |
|  | **60-75y (N=20)** | | **80-89y (N=34)** | | **90-99y (N=26)** | | **100-106y (N=28)** | | | **K-W test^c^** |  |
|  | **Median** | **IQR^a^** | **Median** | **IQR^a^** | **Median** | **IQR^a^** | **Median** | | **IQR^a^** | **p-value** |  |
| **CLU (µg/ml)** | 49.60 | (42.415 - 66.67) | 45.73 | (38.59 - 58.78) | 46.09 | (34.78 - 58.15) | 41.64 | | (31.435 - 53.66) | 0.389 |  |
| **Total Cholesterol (mg/dl)** | 196 | (185 - 226) | 224 | (208 - 250) | 196 | (162 - 226) | 182 | | (160.5 - 200) | <0.0001 |  |
| **HDL (mg/dl)^b^** | 55 | (42.5 - 63) | 55 | (47.5 - 70) | 48 | (38 - 59) | 51 | | (38 - 59) | 0.084 |  |
| **LDL (mg/dl)^b^** | 120.5 | (101.5 - 136.5) | 143.5 | (121.5 - 166.5) | 111.0 | (102 - 142) | 103.0 | | (89 - 130) | 0.0002 |  |
| **Triglycerides (mg/dl)** | 106 | (76 - 171) | 125 | (98 - 161) | 123 | (98 - 141) | 111 | | (90.5 - 156) | 0.673 |  |
| ^a^ IQR, interquartile range  ^b^HDL, high density lipoprotein cholesterol; LDL, low density lipoprotein cholesterol  ^c^K-W = Kruskal Wallis test | | | | | | | |  | | | |
